# Examining How Gender, Race/Ethnicity, and Clinical Roles Moderate the Association Between Sleep and Burnout

**DOI:** 10.1016/j.acepjo.2024.100004

**Published:** 2025-01-08

**Authors:** Tsion Firew, Maody Miranda, Nakesha Fray, Alvis Gonzalez, Alexandra M. Sullivan, Diane Cannone, Joseph E. Schwartz, Jordan F. Karp, Bernard P. Chang, Ari Shechter

**Affiliations:** 1 Department of Emergency Medicine, Columbia University Irving Medical Center, New York, New York, USA; 2 Center for Behavioral Cardiovascular Health, Department of Medicine, Columbia University Irving Medical Center, New York, New York, USA; 3 Department of Psychiatry and Behavioral Health, Renaissance School of Medicine, Stony Brook, New York, USA; 4 Department of Psychiatry, College of Medicine, University of Arizona and University of Arizona PRIDE Program, Tucson, Arizona, USA

**Keywords:** sleep, burnout, healthcare worker, gender, race, ethnicity

## Abstract

**Objectives:**

Sleep disturbance and burnout are common in emergency department health care workers (HCWs), and the 2 are linked. This cross-sectional study evaluated whether gender, race/ethnicity, and clinical roles moderate the association between sleep quality and burnout among emergency department HCWs (N = 129).

**Methods:**

Sleep was assessed with the Pittsburgh Sleep Quality Index (Pittsburgh Sleep Quality Index > 5: poor sleep) and Insomnia Severity Index (Insomnia Severity Index > 8: insomnia). The abbreviated Maslach Burnout Inventory-9 assessed the burnout dimensions of emotional exhaustion, depersonalization , and reduced personal accomplishment . Emotional exhaustion > 9 and either (or both) depersonalization > 6 or personal accomplishment < 9 indicated burnout. Logistic regressions were computed for the association of poor sleep and insomnia with burnout for gender, race/ethnicity, and job role separately.

**Results:**

Poor sleep quality, insomnia, and burnout were seen in 64%, 59%, and 24% of participants, respectively. Poor sleep was more frequently reported in Black, Indigenous, and People of Color (BIPOC) HCWs vs non-BIPOC (72.9% vs 52.5%, *P* = .017). Overall, poor (vs not poor) sleep quality was associated with burnout (odds ratio [OR], 3.14; 95% CI, 1.14-8.64). There was a significant poor sleep-burnout relationship in women (OR, 4.52; 95% CI, 1.10-18.60) that was not seen in men. The poor sleep-burnout relationship was significantly stronger in attending physicians (OR, 6.92; 95% CI, 1.44-33.24) vs registered nurses (OR, 0.28; 95% CI, 0.03-2.30; *P* value for group ∗ predictor interaction term = .021).

**Conclusion:**

BIPOC HCWs had worse sleep quality than non-BIPOC HCWs, and the relationship between sleep quality and burnout was affected by gender and clinical role. These findings highlight the importance of person-level factors in the sleep-burnout relationship in HCWs.

**The Bottom Line**

Emergency department (ED) health care workers (HCWs) experience high rates of poor sleep and burnout. This cross-sectional study of N = 129 ED HCWs evaluated if gender, race/ethnicity, and clinical roles moderate the association between sleep and burnout. Poor sleep quality was reported more frequently in Black, Indigenous, and People of Color HCWs vs nonBlack, Indigenous, and People of Color HCWs. Women showed a pronounced relationship between poor sleep quality and burnout that was not similarly seen in men. Finally, the poor sleep-burnout relationship was significantly stronger in attending physicians vs registered nurses. These findings highlight the importance of person-level factors in the sleep-burnout relationship in ED HCWs.

## 1 Introduction

### 1.1 Background

Burnout represents a significant occupational hazard for health care workers (HCWs), and it is characterized by emotional exhaustion (EE), depersonalization (DP; ie, a negative, detached response toward the recipients of one’s care), and diminished feelings of personal accomplishment (PA).^1^ In the wake of the COVID-19 pandemic, the prevalence of HCW burnout had reached levels as high as 60% because of the intense and sustained challenges that the HCWs faced during the pandemic.^2^ However, even before the pandemic, HCW burnout was endemic. For instance, approximately 50% of physicians reported experiencing burnout symptoms, with the highest rates (∼65%) observed among those in emergency medicine.^3^ Although the literature predominantly focuses on physicians and nurses, burnout also affects other HCWs, including medical technicians and clerical staff.

Burnout has ramifications for the physical (eg, cardiovascular risk) and psychological health (eg, depression) of HCWs in addition to the safety and well-being of the patients that they care for.^4^, ^5^, ^6^ The widespread recognition of the burnout crisis and its adverse impacts has prompted both the National Academy of Medicine and the US Surgeon General to publish advisories on addressing the causes, consequences, and treatment of HCW burnout.^7^^,^^8^

Women, as well as Black, Indigenous, and People of Color (BIPOC) HCWs, are at a heightened risk of experiencing burnout compared with men and their non-BIPOC counterparts. This increased risk is attributable to experiences of sexism, racism, and microaggressions in both the workplace and in broader societal contexts.^9^, ^10^, ^11^ Women and BIPOC HCWs encounter workplace discrimination and are also subjected to broader systemic issues such as police brutality and health disparities.^11^ Although burnout has been studied more generally, there is a paucity of data that specifically address burnout and its related factors in women and BIPOC HCWs within emergency departments (EDs).

Women make up over 70% of ED nurses and 40% of ED physicians, whereas 20% of ED nurses and 15% of ED physicians are BIPOC.^12^^,^^13^ Whereas women compose the majority of the nursing workforce, BIPOC HCWs are underrepresented in medicine in general, and there is concern that attrition may be disproportionately higher among BIPOC HCWs.^14^

Several job demand-related factors, such as inadequate staffing, high patient load, lack of professional autonomy, and excessive administrative burden, likely contribute to burnout in HCWs.^15^ Additionally, exploring how individual psychosocial, sociodemographic, and behavioral factors, including sleep, may influence burnout could inform preventive and therapeutic strategies. Combining organizational or system-level interventions with individual-focused approaches has been recommended to mitigate HCW burnout.^16^

### 1.2 Importance

Emerging evidence suggests that poor sleep may contribute to HCW burnout.^17^ Recent evidence shows that sleep likely has a causal relationship with mental health, including depression and anxiety.^18^^,^^19^ In terms of potentially influencing burnout, sleep disturbance is linked to fatigue, heightened reactivity to negative emotions, reduced reactivity to positive stimuli, and lower self-esteem, helping behavior, and empathy toward others.^20^, ^21^, ^22^, ^23^ As a modifiable behavior with well-established treatment approaches, sleep may represent a potential therapeutic target for HCW burnout. However, there is limited evidence exploring sleep in emergency medicine HCWs, a workforce population noted to have higher burnout risk compared with other disciplines.^24^ Thus, in alignment with a recent American Academy of Sleep Medicine position statement highlighting a “pressing need” to evaluate sleep and burnout, we examined how insomnia symptoms and poor sleep quality may be related to burnout symptoms in a diverse sample of ED HCWs.^25^

### 1.3 Goals of This Investigation

Although the association between sleep and burnout has been studied,^25^ there are limited reports on how sleep and burnout differ by gender, race/ethnicity, and clinical role and how these demographic factors may modulate the relationship between sleep and burnout. This study aimed to address this gap by examining burnout rates among women and BIPOC ED HCWs, as well as by investigating the influence of a modifiable behavioral factor, such as (ie, sleep) on burnout. We explored the association between sleep and burnout and assessed how race, gender, and clinical role may affect this relationship.

## 2 Methods

### 2.1 Study Design and Setting

This study was a cross-sectional analysis of a convenience sample of HCWs from EDs at a major urban medical center. The participants completed questionnaires about their demographics, sleep characteristics, and burnout levels. The study was approved by the Columbia University Medical Center institutional review board (IRB#: AAAS7255), and all participants provided informed consent.

### 2.2 Data Collection

The sample consisted of HCWs from 4 EDs within a large academic medical center in New York City. Recruitment was conducted through a combination of email announcements, meetings, and flyers. Data collection occurred from November 2020 to January 2023, and the study topic was highlighted in the recruitment materials.

### 2.3 Measures

Participants completed questionnaires designed to assess sleep quality and insomnia symptoms, utilizing the Pittsburgh Sleep Quality Index (PSQI) and the Insomnia Severity Index (ISI), respectively.^26^^,^^27^ Scores on the PSQI range from 0 to 21, with higher scores indicating worse overall sleep quality. Scores were dichotomized into poor sleep quality (PSQI global score > 5) or not poor sleep (PSQI global score ≤ 5). Scores on the ISI range from 0 to 28, with higher scores indicating worse severity of insomnia symptoms. Scores were dichotomized into the presence of insomnia symptoms of at least mild severity (ISI score ≥ 8) or none (ISI score < 8). Burnout was measured using the abbreviated Maslach Burnout Inventory-9 (aMBI-9) with the following 3 burnout subscales: EE, DP, and reduced PA. Scores were dichotomized with the following cutoffs: EE score > 9 for high EE, DP score > 6 for high DP, and PA score < 9 for low PA. A designation of overall burnout was given for those individuals with high EE combined with high DP or low PA (or both).^28^^,^^29^

Demographic information that was collected included sex assigned at birth, self-identified gender, race, ethnicity, and clinical role. Gender and sex assigned at birth were queried independently on the questionnaire, and all respondents identified as cisgender. Participants indicated their ethnicity (Hispanic/Latino or non-Hispanic Latin) and race (White, Black, Asian, American Indian/Native American, or Hawaiian/Pacific Islander), with options to decline or specify additional categories. Individuals who identified as Black, Asian, Native American, Hispanic, and/or Latino were categorized as BIPOC, and individuals who identified as non-Hispanic White were categorized as non-BIPOC. Clinical roles were selected from options including attending physician, trainee, registered nurse, advanced practice providers (physician assistants and nurse practitioners), allied health professionals (technicians and unit assistants), and administrators.

### 2.4 Analysis

Descriptive statistics were reported as means with SDs or proportions (n, %). Chi-squared tests were utilized to compare the proportions of participants with poor sleep, insomnia, and burnout across gender, race/ethnicity groups, and clinical roles. Logistic regressions were computed for adjusted odds ratios (OR) to determine the association between categoric PSQI (poor sleep quality) and ISI (insomnia symptoms) sleep dimensions (ie, PSQI > 5 vs ≤5 and ISI ≥ 8 vs <8) with the presence of burnout, adjusting for age and broad job title grouping (attendings and registered nurses vs all others). Separate sleep-burnout regressions were conducted for each demographic subgroup (gender, race/ethnicity, and job role). Subgroup analyses for job roles were restricted to attending physicians and registered nurses because of the small sample sizes of other roles. To examine differences in the sleep-burnout relationship between genders, BIPOC status, and job roles, the overall logistic regression model was reestimated, adding the main effect of “group” and the multiplicative interaction term of “group ∗ ‘predictor’” to the equation. The *P* value for the interaction term is reported.

## 3 Results

A summary of participant demographics, as well as sleep and burnout characteristics, is detailed in Table 1. The study included 129 participants, which comprised 81 women (62.8%) and 70 individuals who identified as BIPOC (54.3%). Within the overall sample, 63.6% exhibited poor sleep quality, and 58.9% experienced insomnia. High levels of EE were observed in 42.6% of participants, high DP in 27.1%, reduced PA in 17.1%, and overall burnout was present in 24% of the cohort.

**Table 1:** Demographics of sample, N = 129.

Variable	N (%)
Gender	
Men	48 (37.2)
Women	81 (62.8)
Race/ethnicity	
Non-Hispanic White	59 (45.7)
Non-Hispanic Black	10 (7.8)
Non-Hispanic Asian	28 (21.7)
Hispanic/Latino	23 (17.8)
Other/more than 1 race	5 (3.9)
Unknown/declined to answer	4 (3.1)
BIPOC status	
Non-BIPOC	59 (45.7)
BIPOC	70 (54.3)
Clinical role	
Attending physician	57 (44.2)
Trainee^a^	9 (7.0)
Registered nurse	29 (22.5)
Advanced practice provider^b^	16 (12.4)
Allied health professional^c^	16 (12.4)
Administrator	2 (1.6)
Poor sleep (PSQI > 5)	82 (63.6)
Insomnia (ISI ≥ 8, mild or worse)	76 (58.9)
aMBI-9	
EE (subscale > 9)	55 (42.6)
DP (subscale > 6)	36 (27.1)
PA (subscale < 9)	22 (17.1)
Burned out (EE > 9 and [DP > 6 or PA < 9])	31 (24.0)

	**Mean (SD)**
Age, y	41.0 (10.0)
PSQI global score	7.2 (3.1)
ISI score	8.4 (4.6)
aMBI-9 component scores	18.8 (9.3)
EE subscale	8.3 (4.5)
DP subscale	4.7 (4.2)
PA subscale	12.2 (3.7)

aMBI-9, abbreviated Maslach Burnout Inventory-9; BIPOC, Black, Indigenous, and People of Color; DP, depersonalization; EE, emotional exhaustion; ISI, Insomnia Severity Index; PA, personal accomplishment; PSQI, Pittsburgh Sleep Quality Index.

a Trainee: resident and fellow.

b Advanced practice provider: nurse practitioner and physician assistant.

c Allied health professional: social worker, patient navigator, and care coordinator.

Table 2 shows the variations in sleep and burnout parameters according to gender, race/ethnicity, BIPOC status, and clinical role. Gender and sex were queried independently on the questionnaires, with all respondents identifying as cisgender. Consequently, the terms “men” and “women” in this context align with the sex assigned at birth. The analysis revealed no significant differences in the prevalence of poor sleep, insomnia, or burnout between men and women. However, poor sleep quality was significantly more prevalent among BIPOC HCWs compared with non-BIPOC HCWs (72.9% vs 52.5%, *P* = .017). Conversely, insomnia and burnout rates did not show significant differences between BIPOC and non-BIPOC groups. The presence of disturbed sleep and burnout did not differ significantly between job roles.

**Table 2:** Percentage of those with disturbed sleep, insomnia, and burnout, by subgroup.

Variable	Poor sleep (PSQI > 5) %	Insomnia (ISI ≥ 8) %	Burned out^a^ %
Gender			
Men	64.6	58.3	27.1
Women	63.0	59.3	22.2
*P* value	.85	.92	.53
Race/ethnicity			
Non-Hispanic White	52.5	59.3	22.0
Non-Hispanic Black	70.0	60.0	30.0
Non-Hispanic Asian	78.6	67.9	32.1
Hispanic/Latino	69.6	52.2	26.1
Other/more than 1 race	60.0	40.0	0.00
Unknown/declined to answer	75.0	50.0	0.00
*P* value	.25	.82	.52
BIPOC status			
Non-BIPOC	52.5	59.3	22.0
BIPOC	72.9	58.6	25.7
*P* value	.017^e^	.93	.63
Job title/role			
Attending physician	57.9	64.9	26.3
Trainee^b^	77.8	44.4	22.2
Registered nurse	75.9	69.0	24.1
Advanced practice provider^c^	50.0	56.2	18.8
Allied health professional^d^	68.8	31.2	18.8
Administrator	50.0	50.0	50.0
*P* value	.41	.16	.92

BIPOC, Black, Indigenous, and People of Color; ISI, Insomnia Severity Index; PSQI, Pittsburgh Sleep Quality Index.

a Calculated from abbreviated Maslach Burnout Inventory subscales: emotional exhaustion subscale > 9 and either (or both) depersonalization subscale > 6 or personal accomplishment subscale < 9.

b Trainee: resident and fellow assistant.

c Advanced practice provider: nurse practitioner and physician assistant.

d Allied health professional: social worker, patient navigator, and care coordinator.

e Statistically significant *P* value (*P* < .05).

Table 3 presents the results of logistic regressions predicting burnout from sleep parameters, separately by gender, BIPOC status, and job roles. In the overall sample, those with poor sleep quality demonstrated a 3-fold increase in odds of burnout compared with those without poor sleep quality (OR, 3.14; 95% CI, 1.14-8.64). In women, those with poor sleep quality demonstrated a 4.5-fold increase in odds of burnout compared with those without poor sleep quality (OR, 4.51; 95% CI, 1.10-18.60), although this relationship was not significant in men (OR, 1.92; 95% CI, 0.43-8.61). The relationships between the presence of insomnia and burnout were not significant for either men or women. In the BIPOC status subanalyses, the relationships between sleep disturbance and burnout were not significant for either BIPOC or non-BIPOC participants. Notably, a significant relationship between poor sleep and burnout was identified among attending physicians, with those physicians with poor sleep quality demonstrating close to a 7-fold increase in odds of burnout compared with those without poor sleep quality (OR, 6.92; 95% CI, 1.44-33.24), but this relationship was not seen among registered nurses (OR, 0.28; 95% CI, 0.03-2.30). Furthermore, the strength of the poor sleep-burnout relationship was significantly different between attending physicians and registered nurses (*P* = .021).

**Table 3:** ORs predicting burnout from sleep parameters.

Variable	Predictor			
	Poor sleep		Insomnia	
	OR (95% CI)	*P* value	OR (95% CI)	*P* value
Full sample	3.14 (1.14-8.64)	.027^a^	1.66 (0.68-4.09)	.27
By gender				
Men	1.92 (0.43-8.61)	.39	1.07 (0.28-4.10)	.93
Women	4.51 (1.10-18.60)	.037^a^	2.34 (0.69-7.93)	.17
*P* value of difference		.42		.39
By BIPOC status				
Non-BIPOC	2.68 (0.68-10.51)	.16	1.15 (0.31-4.28)	.84
BIPOC	3.45 (0.69-17.20)	.13	2.17 (0.63-7.44)	.22
*P* value of difference		.81		.48
By job title/role				
Attending physician	6.92 (1.44-33.24)	.016^a^	1.46 (0.39-5.46)	.58
Registered nurse	0.28 (0.03-2.30)	.23	2.64 (0.26-27.17)	.41
*P* value of difference		.021^a^		.66

Disturbed sleep: Pittsburgh Sleep Quality Index total score > 5.

Insomnia: Insomnia Severity Index ≥ 8 (mild, moderate, or severe insomnia).

Each analysis controls for age and broad job title grouping (attendings and registered nurses vs all others). The *P* value of the difference indicates the group ∗ predictor interaction term that compares the strength of the associations between groups.

BIPOC, Black, Indigenous, and People of Color; OR, odds ratio.

a Statistically significant *P* values (*P* < .05).

## 4 Limitations

Despite the relevance of these findings, the study’s limited sample size affects generalizability and statistical power. Because of the limited number of other health care professionals beyond nurses and doctors, we focused the analyses on the clinical role’s impact on these 2 groups. The cross-sectional design precludes conclusions about the directionality of effects (eg, if poor sleep contributes to burnout or vice versa). However, we report a novel finding that gender and job role may moderate the sleep-burnout relationship, suggesting that solutions to address burnout among ED HCWs could be tailored based on individual characteristics. Additionally, the mention of the study topic in the recruitment flyers might have introduced a selection bias, potentially attracting individuals who have strong opinions on the topic. However, given the widespread and pervasive nature of burnout among HCWs at the time, we leveraged this to encourage participant enrollment. Anecdotally, participants were very eager to participate in research to help understand the causes underlying burnout in HCWs. Finally, it is important to note that the study utilized self-reported measures (ie, surveys) to assess sleep and burnout. Nonetheless, we employed 2 validated and widely accepted instruments for measuring sleep quality and insomnia symptoms, in addition to a multidimensional assessment tool for burnout, rather than relying on single-item measures that are commonly used in the literature.

## 5 Discussion

To our knowledge, this is the first study to evaluate how gender, BIPOC status, and job role moderate the relationship between sleep disturbances and burnout in ED HCWs. Our findings indicate that poor sleep quality is significantly associated with burnout and that gender and clinical role may affect this relationship.

Our results corroborate existing literature highlighting high prevalence rates of sleep disturbances and burnout among HCWs.^25^ Specifically, approximately 60% participants reported poor sleep quality and insomnia, whereas nearly a quarter (24%) demonstrated burnout. Among burnout symptoms, high EE was the most prevalent, followed by high DP and reduced PA. These findings contribute to the growing body of evidence linking sleep disturbances to burnout in HCWs.^24^^,^^30^

Gender-based comparisons revealed that poor sleep quality had an association with burnout among women but not men. This is particularly relevant given that women are known to be at higher risk for both sleep disturbances and burnout.^10^^,^^31^ The observed differences suggest that there may be unique psychosocial, biological, or other factors associated with women that contribute to an increased risk of the adverse effects of sleep disturbance on burnout. Future research should investigate these potential factors at the individual, group, and systemic levels to better understand women’s susceptibility to these adverse outcomes.

Regarding BIPOC status, BIPOC HCWs exhibited higher rates of poor sleep quality compared with their non-BIPOC counterparts. Although burnout was also more prevalent among BIPOC (25.7%) compared with non-BIPOC (22%), the difference was not statistically significant. These findings align with trends observed in other medical fields where Hispanic and Black HCWs report severe burnout symptoms.^32^ Historically underrepresented minority groups in medicine, such as Black and Hispanic groups, exhibit worse sleep quality and increased burnout symptoms compared with their White counterparts.^10^^,^^32^ This disparity is important given that attrition rates among BIPOC/underrepresented minority groups are notably higher compared with Whites. This underscores the need for improved retention strategies within the medical field, not just recruitment.^33^

An examination of clinical roles revealed that registered nurses had higher rates of poor sleep quality compared with attending physicians (75.9% vs 57.9%). Previous studies suggest that longer working hours and the physically demanding nature of nursing roles may contribute to these observations.^34^^,^^35^ Furthermore, differences in compensation and socioeconomic status between physicians and nurses could exacerbate mental health challenges among nurses. This indicates that addressing these disparities may be crucial for improving overall health outcomes among nurses.

Although existing research has established a general link between poor sleep quality and burnout, our study introduces novel insights by demonstrating that the relationship between these factors is moderated by gender and clinical role in ED HCWs.^17^^,^^36^ Specifically, the relationship between poor sleep and burnout is present in women but not men, and the impact of poor sleep on burnout is stronger in attending physicians compared with nurses. Thus, despite the higher proportion of women in the role, individuals choosing to work in ED nursing may exhibit increased resilience to the adverse effects of sleep disturbances on burnout. Consequently, the relatively worse sleep quality in nurses compared with attending physicians did not result in higher burnout symptoms in the current sample. This finding suggests a need for further investigation into factors influencing sleep and burnout (eg, resilience) among HCWs. Although peer support services, like those offered through Columbia University Medical Center’s Cope Columbia (our home institution), provide valuable short-term assistance and resilience-building, addressing sleep disturbances and burnout requires enduring and fundamental policy changes.^37^ Future research should examine individual, group-level, and system-level factors that may influence HCWs’ vulnerability to these psychological and physiological outcomes.

In summary, to our knowledge, this study is the first to explore how gender, race/ethnicity, and clinical role impact the relationship between sleep disturbances and burnout among ED HCWs. Our findings indicate that poor sleep quality is linked to increased burnout, with a pronounced effect observed in women. Additionally, BIPOC HCWs reported worse sleep quality compared with their non-BIPOC counterparts, and registered nurses experienced poorer sleep than attending physicians. Building on these findings, future research should further elucidate the pathways through which demographic factors, such as gender, contribute to mental health outcomes in HCWs. This may involve examining the unique individual-level stressors encountered by these professionals. Such insights could inform the development of system-level interventions, such as reducing work hours or implementing circadian-friendly shift schedules, to enhance sleep quality and overall well-being within the health care workforce, thereby supporting both professional efficacy and excellence in patient care.

## Author Contributions

T.F., B.P.C., and A.S. conceived the study, designed the trial, and obtained research funding. T.F., M.M., A.G., A.S., and D.C. supervised the conduct of the trial and data collection. T.F., B.P.C., M.M., A.G., A.S., and D.C. undertook the recruitment of participants and managed the data, including quality control. N.F. and J.E.S. provided statistical advice on study design and analyzed the data. B.P.C. and A.S. chaired the data oversight committee. T.F. drafted the manuscript, and all authors contributed substantially to its revision. T.F. takes responsibility for the paper as a whole.

## Funding and Support

This work was supported by the 10.13039/100000002National Institutes of Health (grants R01HL146911 and R01HL157341 to A.S. and B.P.C. and R25HL126140 PRIDE to T.F.).

The funder had no role in the design and conduct of the study; collection, management, analysis, and interpretation of the data; preparation, review, or approval of the manuscript; and decision to submit the manuscript for publication.

## Data Availability

Partial or complete datasets and data dictionaries are available from the date of publication on request to Dr Tsion Firew via tsionfirew@gmail.com to investigators who provide an IRB letter of approval.

## Conflict of Interest

Dr Chang reported receiving stock options from Mighty Health Digital Health wellness application for serving as an adviser outside the submitted work. No other disclosures were reported.
